# Analyzing natural herd immunity media discourse in the United Kingdom and the United States

**DOI:** 10.1371/journal.pgph.0000078

**Published:** 2022-01-12

**Authors:** Marco Zenone, Jeremy Snyder, Alessandro Marcon, Timothy Caulfield

**Affiliations:** 1 Faculty of Public Health & Policy, London School of Hygiene and Tropical Medicine, London, United Kingdom; 2 Faculty of Health Sciences, Simon Fraser University, Burnaby, British Columbia, Canada; 3 Health Law Institute, University of Alberta, Edmonton, Alberta, Canada; The University of British Columbia, CANADA

## Abstract

Natural herd immunity, where community-acquired infections in low-risk populations are used to protect high risk populations from infection–has seen high profile support in some quarters, including through the Great Barrington Declaration. However, this approach has been widely criticized as ineffective and misinformed. In this study, we examine media discourse around natural herd immunity in the United States (US) and United Kingdom (UK) to better understand how this approach was promoted. Country-specific news media publications between March 11, 2020 and January 31, 2021 were searched for references to herd immunity. News articles focused on herd immunity and including a stakeholder quote about herd immunity were collected, resulting in 400 UK and 144 US articles. Stakeholder comments were then coded by name, organization, organization type, and concept agreement or disagreement. Government figures and a small but vocal coalition of academics played a central role in promoting natural herd immunity in the news media whereas critics were largely drawn from academia and public health. These groups clashed on whether: natural herd immunity is an appropriate and effective pandemic response; the consequences of a lockdown are worse than those of promoting herd immunity; high-risk populations could be adequately protected; and if healthcare resources would be adequate under a herd immunity strategy. False balance in news media coverage of natural herd immunity as a pandemic response legitimized this approach and potentially undermined more widely accepted mitigation approaches. The ability to protect high risk populations while building herd immunity was a central but poorly supported pillar of this approach. The presentation of herd immunity in news media underscores the need for greater appreciation of potential harm of media representations that contain false balance.

## Introduction

Natural herd immunity–understood as indirect protection from an infectious disease due to sufficient immunity acquired by previous infection within the local community–has emerged as a containment strategy during the COVID-19 pandemic [1]. Adopting a natural herd immunity strategy in the context of COVID-19 requires that policymakers and public health officials allow populations considered at low-risk of serious harm from COVID-19 –such as young people–to be exposed to potential infection while protecting those considered high risk. These infections are then thought to lead to immunity which, on a population scale, infers protection for other high-risk groups such as seniors and those with other medical conditions.

The Great Barrington Declaration, penned October 4^th^ 2020 by a small but highly visible group of scientists based in the United Kingdom (UK) and United States (US), supports implementing this approach [2]. The authors of the declaration argue that “[t]hose who are not vulnerable should immediately be allowed to resume life as normal” to build up immunity across the population. Based on this and other forms of advocacy for natural herd immunity, government officials in the United States [3], United Kingdom [4], Sweden [5], and elsewhere have adopted or advocated for features of this response at certain stages of the pandemic.

Natural herd immunity as a pandemic response has been condemned by most public health institutions and academics [6, 7]. Critics such as the World Health Organization (WHO) argue that the death toll from a herd immunity approach would be intolerable and overwhelm healthcare systems [8, 9]. For example, Ilesanmi and colleagues estimated the predicted deaths from the West African population acquiring COVID-19 needed in order to achieve herd immunity was 5.2 million deaths [10]. Scientists warn that it isn’t impossible to control the spread of the virus to certain low-risk populations [11]. It also isn’t clear who is at higher risk of COVID-19 –for example, young people may get a persistent illness [12]. Researchers also note that herd immunity is likely an ineffective strategy due to possibility of reinfection and mutant strains [13]. In countries where a natural herd immunity was implemented, such as Sweden, mortality and infections are significantly higher than in other, demographically similar locales [14]. Similarly, herd immunity has not developed in areas where the majority of the population contracted the virus, such as in Manaus, Brazil [15].

The public discourse around natural herd immunity as a response to the COVID-19 pandemic has echoes in misinformation around purported treatments for the coronavirus and the safety and the efficacy of vaccines [16]. This paper investigated the media discourse of two countries that were criticized for strategies described as allowing certain populations to be infected with COVID-19, the United States (US) and United Kingdom (UK), to determine the kinds of rationale used to support such measures and how media reporting covered the debate for the public. We determined the societal actors, organizations, special interest groups, networks, and coalitions representing herd-immunity as an effective COVID-19 containment strategy. Second, we assessed the concepts and messaging strategy used advocate for herd immunity to governments and the public. Last, we determined the amount of media coverage those advocating for, or against, a natural herd immunity approach received, including by specific argument.

## Materials and methods

To identify herd immunity news coverage in the UK and US, searches were conducted on February 6^th^, 2020 and completed on FACTIVA–an international news database [17]. We selected FACTIVA due to its search features, such as consistency in searching across countries, and its extensive, continuously updated news sources. The database was also created by reputable news sources such as Reuters. The search phrase used was [herd immunity OR “herd-immunity”] and applied to the title or first paragraph of news articles published between the dates of March 11^th^, 2020 and January 31^st^, 2021. We did not search full text for reference to the search terms due to project feasibility and because we sought articles focused on reporting or commentary specifically related to herd immunity. We selected these dates to reflect the WHO pandemic declaration [18] and included articles up until the week prior to the search. Articles were restricted only to publications available in English-language. The search identified 927 articles for the UK and 537 articles for the US. All articles were then exported into individual PDF files for inclusion review. Article details, such as title, date published, author, and publication, were exported in an excel file to organize the inclusion review process.

To decide inclusion, each article was reviewed by the first author and needed to meet the following criteria: (1) herd-immunity is a primary focus of the article; (2) the article contains at least one direct quotation from stakeholder or quote attributable to a specific person or organization relating to herd immunity; and (3) is a news article or editorial and not a letter to the editor or blog. The first author flagged and consulted with the second author when an inclusion decision was unclear. The second author also audited 25% of all included articles to ensure consistent inclusion criteria application. All decisions, auditing, and comments between the first and second author were recorded on the project spreadsheet database. After this process, 400 articles were included from the UK and 144 from the US.

The included articles were then converted to text files and uploaded to the Discourse Network Analyzer (DNA) application [19]. Each author then reviewed a subset of articles to determine the principal concepts under debate in media reporting. After an iterative discussion determining possible concepts to examine, all authors agreed to code for agreement or disagreement to 4 defined concept statements. Stakeholder comments were coded by name, organization, stakeholder type (definitions provided in Table 1) and concept agreement or disagreement. Stakeholder types were identified in the iterative manner of concept identification. While certain actors can belong to multiple categories, the authors consulted and selected the category in which stakeholder type best fit description of their primary occupation in their specific comment context. All author disagreements were resolved through discussion. To ensure consistent code application, the second author audited approximately 50% of final coding. Data is provided in the S1 and S2 Datasets. To visualize coding, two-mode network country-specific data (stakeholder by concept) was exported to the network analysis software Visone [20]. Each concept by country was then visualized through grouping of disagreement or agreement and applying specific colors to stakeholders to represent stakeholder type. The authors then reviewed the frequency totals by stakeholder and concept agreement or disagreement, as well as the graphs, to determine trends in natural herd immunity debate and media coverage.

**Table 1:** Stakeholder type definitions.

Stakeholder Type	Definition
**Academic**	Primary occupational attachment is an academic institution such as a university, college, think tank, research institute, or research-intensive organization.
**Business**	Primary occupational attachment is a for-profit business entity.
**Government**	Employed by a local, regional, or national government entity in established and ongoing role but not elected to the position. Does not include health-related advisors if advice is offered from peripheral public health-related organizations, agencies, or higher education institution.
**Individual**	Persons not attached to any specific stakeholder type in media reporting.
**News/Media**	Primary occupational attachment is a news, media, or knowledge dissemination organizations such as television, radio, or journalism.
**Political**	Persons elected to local, regional, national government public office.
**Public Health/Medicine**	Primary occupational attachment is to a public health-related organization or health treatment organization such as a hospital or public health clinic.
**Union**	Primary occupational attachment is an industry-representing union.

## Results

### Overview

Among articles retrieved in the UK, a total of 1243 statements agreeing or disagreeing with a defined concept related to herd immunity were recorded from 148 persons/groups, representing 93 organizations. Out of 400 articles from the UK, 48·8% (n = 195) included statements agreeing that herd immunity is a viable approach and 75·3% (n = 301) included statements disagreeing. Among articles retrieved in the US, a total of 704 statements agreeing or disagreeing with a defined concept were recorded from 131 persons/groups, representing 83 organizations. Out of 144 articles from the US, 50·0% (n = 72) included statements agreeing that herd immunity is a viable approach and 88·2% (n = 127) included statements disagreeing. Tables 2 and 3 outline country-specific code application by stakeholder type and concept coding agreement.

**Table 2:** Overview of UK natural herd immunity discourse media debate by stakeholder type.

Stakeholder Type	Natural herd immunity is an appropriate or efficacious COVID-19 control strategy/natural herd immunity working or being achieved		Consequences of lockdown/related control m5easures exceed consequences of herd immunity approach		At risk populations can be protected in herd immunity approach		Healthcare services and capacity can be managed in herd immunity approach	
	Disagree	Agree	Disagree	Agree	Disagree	Agree	Disagree	Agree
**Academic**	197 (66.6%)	99 (33.4%)	51 (46.8%)	58 (53.2%)	50 (40.7%)	73 (59.3%)	16 (72.7%)	6 (27.3%)
**Government**	36 (22.5%)	124 (77.5%)	6 (50.0%)	6 (50.0%)	7 (15.6%)	38 (84.4%)	5 (31.3%)	11 (68.8%)
**Political**	117 (96.7%)	4 (3.3%)	21 (91.3%)	2 (8.7%)	17 (89.5%)	2 (10.5%)	9 (100.0%)	0 (0.0%)
**Public Health/Medical**	106 (96.3%)	4 (3.7%)	28 (90.3%)	3 (9.7%)	11 (91.7%)	1 (8.3%)	4 (100.0%)	0 (0.0%)
**Journalism/Media**	32 (71.1%)	13 (28.9%)	13 (59.1%)	9 (40.9%)	6 (54.5%)	5 (45.5%)	3 (75.0%)	1 (25.0%)
**Individual**	8 (47.1%)	9 (52.9%)	3 (100.0%)	0 (0.0%)	0 (0.0%)	2 (100.0%)	2 (100.0%)	0 (0.0%)
**Business**	4 (33.3%)	8 (66.7%)	1 (33.3%)	2 (66.7%)	0 (0.0%)	7 (100.0%)	1 (100.0%)	0 (0.0%)
**Union**	2 (100.0%)	0 (0.0%)	0 (NA)	0 (NA)	0 (NA)	0 (NA)	0 (NA)	0 (NA)
**Total**	**502 (65.8%)**	**261 (34.2%)**	**123 (60.6%)**	**80 (39.4%)**	**91 (41.6%)**	**128 (58.4%)**	**40 (69.0%)**	**18 (31.0%)**

**Table 3:** Overview of US natural herd immunity discourse media debate by stakeholder type.

Stakeholder Type	Natural herd immunity is an appropriate or efficacious COVID-19 control strategy/natural herd immunity working or being achieved		Consequences of lockdown/related control measures exceed consequences of herd immunity approach		At risk populations can be protected in herd immunity approach		Healthcare services and capacity can be managed in herd immunity approach	
	Disagree	Agree	Disagree	Agree	Disagree	Agree	Disagree	Agree
**Academic**	147 (73.9%)	52 (26.1%)	69 (74.2%)	24 (25.8%)	37 (49.3%)	38 (50.7%)	14 (100.0%)	0 (0.0%)
**Public Health/Medical**	64 (100.0%)_	0 (0.0%)	25 (100.0%)	0 (0.0%)	11 (100.0%)	0 (0.0%)	1 (100.0%)	0 (0.0%)
**Government**	27 (45.8%)	32 (54.2%)	1 (10.0%)	9 (90.0%)	1 (4.0%)	24 (96.0%)	1 (16.7%)	5 (83.3%)
**Political**	15 (45.5%)	18 (54.5%)	2 (66.7%)	1 (33.3%)	0 (0.0%)	6 (100.0%)	0 (NA)	0 (NA)
**Journalism/Media**	11 (68.8%)	5 (31.3%)	8 (88.9%)	1 (11.1%)	4 (80.0%)	1 (20.0%)	2 (100.0%)	0 (0.0%)
**Individual**	10 (76.9%)	3 (23.1%)	10 (90.9%)	1 (9.1%)	3 (75.0%)	1 (25.0%)	2 (100.0%)	0 (0.0%)
**Business**	4 (66.7%)	2 (33.3%)	4 (66.7%)	2 (33.3%)	3 (60.0%)	2 (40.0%)	1 (100.0%)	0 (0.0%)
**Total**	**278 (71.3%)**	**112 (28.7%)**	**119 (75.8%)**	**38 (24.2%)**	**59 (45.0%)**	**72 (55.0%)**	**21 (80.8%)**	**5 (19.2%)**

### Concept #1: Natural herd immunity is an appropriate of efficacious COVID-19 control strategy/natural herd immunity is working or being achieved

Academic, government, political figures, public health, and journalist or media stakeholder types were considerably more represented in the media than individuals, businesses, and unions (Tables 2 and 3). Media reporting of public health stakeholder comments in both areas showed no conflicting views and near universal agreement that natural herd immunity is an ineffective COVID-19 mitigation strategy. Notably, media coverage of comments from the WHO Director-General, Dr. Tedros Adhanom Ghebreyesus, demonstrated public health agreement against a natural herd immunity approach, stating that “[a]llowing a dangerous virus that we don’t fully understand to run free is simply unethical. It’s not an option” and “[n]ever in the history of public health has herd immunity been used as a strategy for responding to an outbreak.” These comments are similar to those made by Dr. Anthony Fauci, Director of the National Institute of Allergy and Infectious Diseases (US), who made frequent comments denouncing herd immunity, stating: “[w]e’re very concerned about this concept of letting people get infected, or letting herd immunity coming in” and “[w]e certainly are not wanting to wait back and just let people get infected so that you can develop herd immunity. That’s certainly not my approach.”

**Fig 1:**
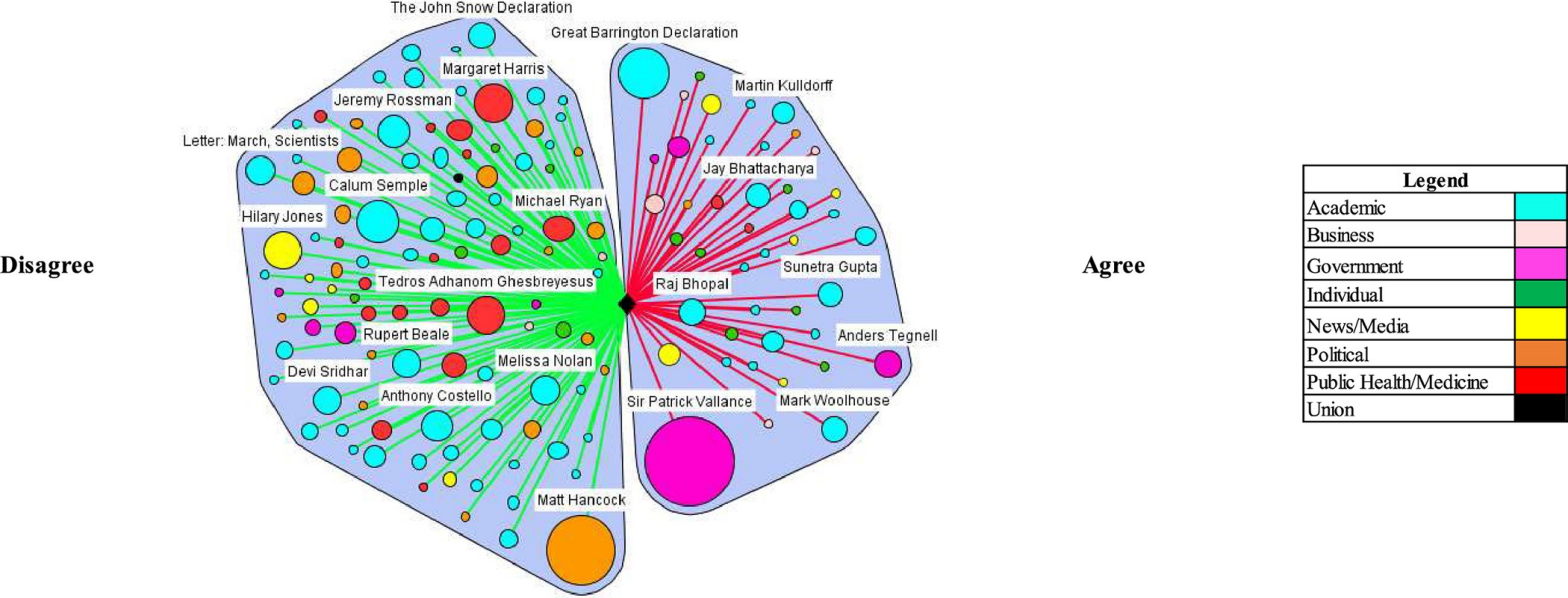
Natural herd immunity is an appropriate or efficacious COVID-19 control strategy/natural herd immunity is working or being achieved (UK).

**Fig 2:**
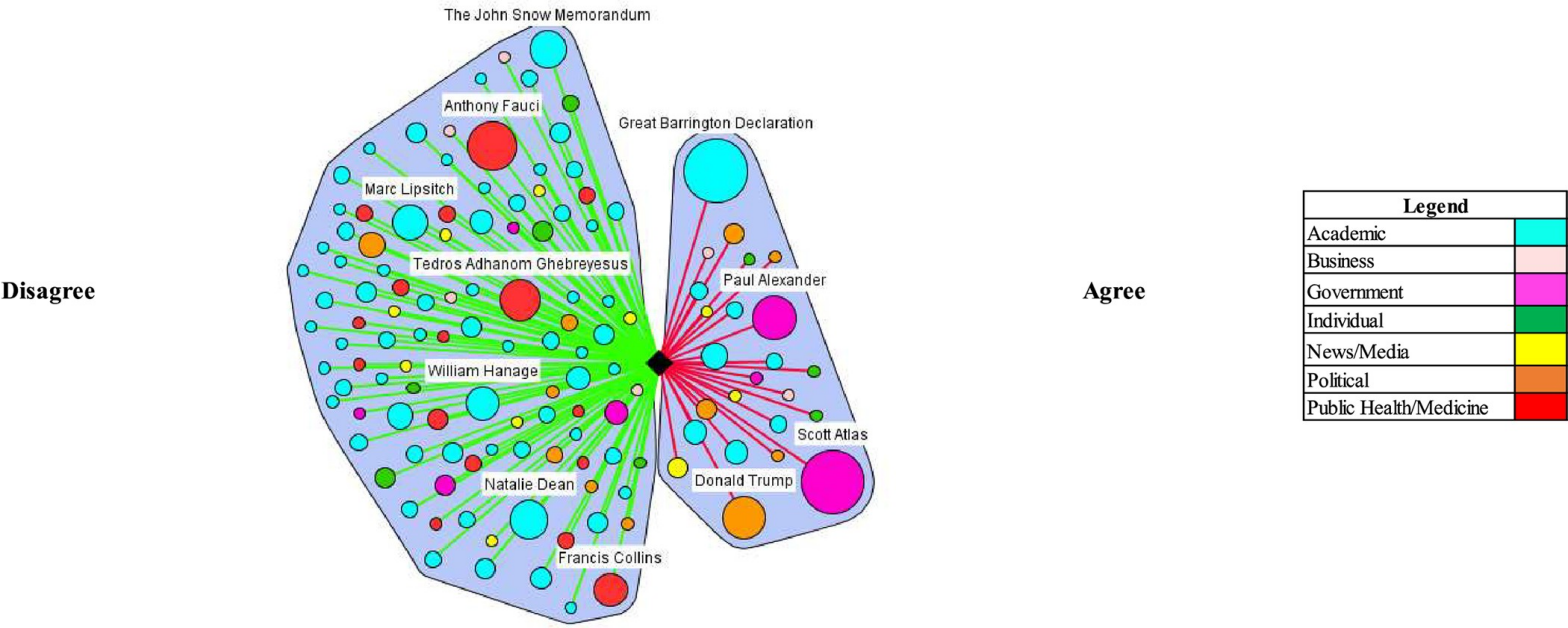
Natural herd immunity is an appropriate or efficacious COVID-19 control strategy/natural herd immunity is working or being achieved (US).

In both the UK and US, media coverage demonstrated elements of false balance in reporting among government and academic stakeholder types. False balance is the media portrayal of an issue where contesting perspectives are presented as balanced or equally valid, contrary to the supportive evidence. (e.g. vaccines cause autism) [21]. False balance was especially evident among government stakeholder types in the US and partially evident among academics in the UK. In both countries, the media portrayal of academics’ perspectives leaned toward disagreeing with herd immunity, but in the UK, government stakeholder perspectives were heavily portrayed as agreeing (Tables 2 and 3). Various stakeholders contributed this discourse (Figs 1 and 2), which notably included, Sir Patrick Vallance, the Government Chief Scientific Advisor–who advocated for herd immunity by stating on March 13^th^, 2020 “our aim is to try and reduce the peak, broaden the peak, not suppress it completely. Also, because the vast majority of people get a mild illness [and] build up some kind of herd immunity, more people are immune to this disease and we reduce the transmission.” In the US, Dr. Scott Atlas, an advisor on COVID-19 to President Donald Trump, was an influential figure making similar comments: “[w]e like the fact that there’s a lot of cases in low-risk populations because that’s exactly how we’re going to get herd immunity.” Another official appointed by the Trump Administration, Dr. Paul Alexander, had emails released that showed he advocated to allow low risk groups to contract the virus: “we want them infected… and recovered…. with antibodies.”

The critical or countering herd immunity perspectives in the media were most evident in both contexts by academics (Figs 1 and 2). Notably, a coalition of scientists against natural herd immunity wrote the *The John Snow Memorandum* which labelled herd immunity a “dangerous fallacy unsupported by scientific evidence.” The exception to academic support against a herd immunity approach was a small, but vocal coalition of scientists–Drs. Martin Kulldorf, Jay Battacharya, and Sunetra Gupta–who authored an influential and heavily publicized petition calling on governments to adopt a herd immunity approach known as the Great Barrington Declaration, and argued: “those who are at minimal risk of death to live their lives normally to build up immunity to the virus through natural infection, while better protecting those who are at the highest risk.” In the UK, Matt Hancock, the Secretary of State for Health and Social Care, received considerable media attention, stating that that the UK was not following a policy of herd immunity in response to the comments made by Vallance: “herd immunity is not our policy, it’s not our goal. Our goal is to protect life and our policy is to fight the virus and protect the vulnerable and the [National Health Service].”

### Concept #2: Consequences of lockdown/related control measures exceed consequences of herd immunity approach

**Fig 3:**
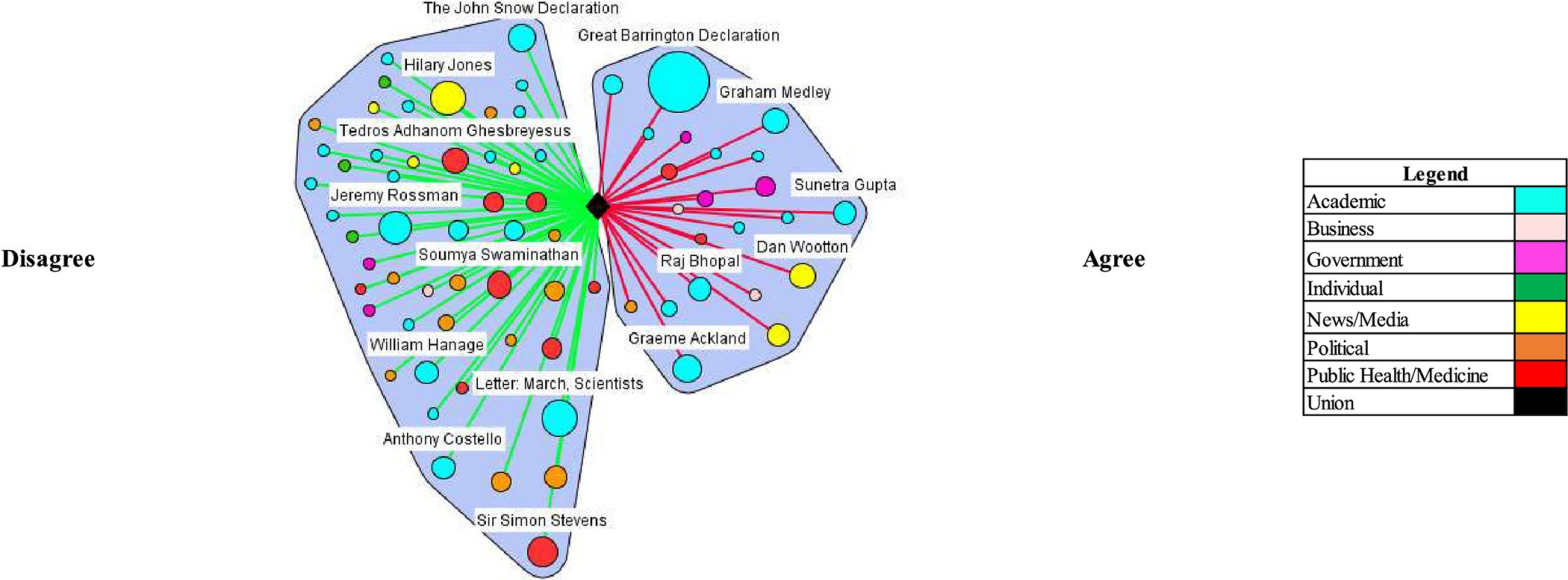
Consequences of lockdown/related control measures exceed consequences of herd immunity approach (UK).

**Fig 4:**
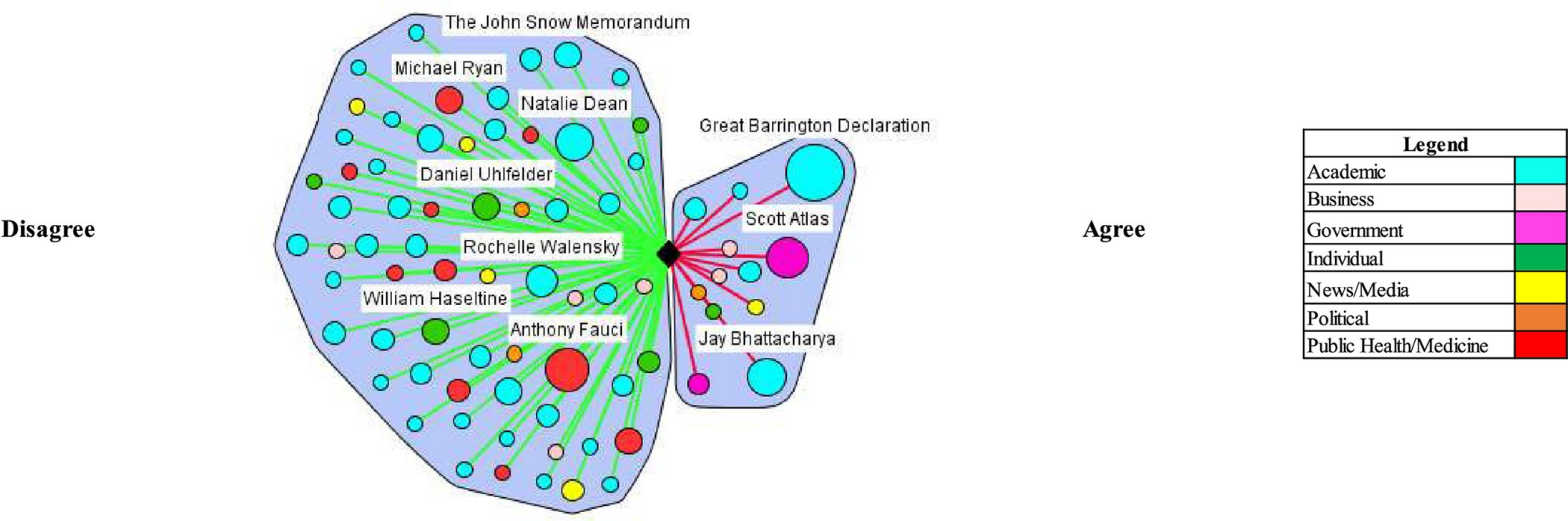
Consequences of lockdown/related control measures exceed consequences of herd immunity approach (US).

Herd immunity proponents argued that the benefits of a herd immunity approach outweighed the consequences if proper precautions were adopted. Specifically, they argued that pandemic mitigation measures such as lockdowns cause more damage than COVID-19. The most frequent stakeholder making statements in the UK and the US supporting this view (Figs 3 and 4)–the Great Barrington Declaration–stated: “As infectious disease epidemiologists and public health scientists we have grave concerns about the damaging physical and mental health impacts of the prevailing Covid-19 policies” and that “[k]eeping these measures in place until a vaccine is available will cause irreparable damage.” The specific harms mentioned include “lower childhood vaccination rates, worsening cardiovascular disease outcomes, fewer cancer screenings and deteriorating mental health–leading to greater excess mortality in years to come.” Certain reporting included comments from Atlas indicating that not adopting a herd immunity approach might extend the pandemic length and its harm.

The majority of actors commented that the consequences of a herd immunity approach would lead to excess death and suffering, thus making it unacceptable. Fauci vocally disagreed with the assessments of the Great Barrington Declaration authors and Atlas, commenting that if the disease were allowed to spread it would lead to unacceptable levels of mortality and suffering: “[i]f already, 200,000 people have died and you want to let things go to herd immunity, you’re going to get a lot of suffering and a lot deaths” and that the “death would be enormous and totally unacceptable.” The outcome of other countries undertaking a natural herd immunity approach–such as Sweden and their failures were commented on by figures such as Dr. Natalie Dean, an assistant professor at the University of Florida, Others, such as Dr. Jeremy Rossman, a virology lecturer at the University of Kent, warned that a herd immunity approach does not fully consider the suffering or impact on quality of life.

### Concept #3: At risk populations can be protected in herd immunity approach

**Fig 5:**
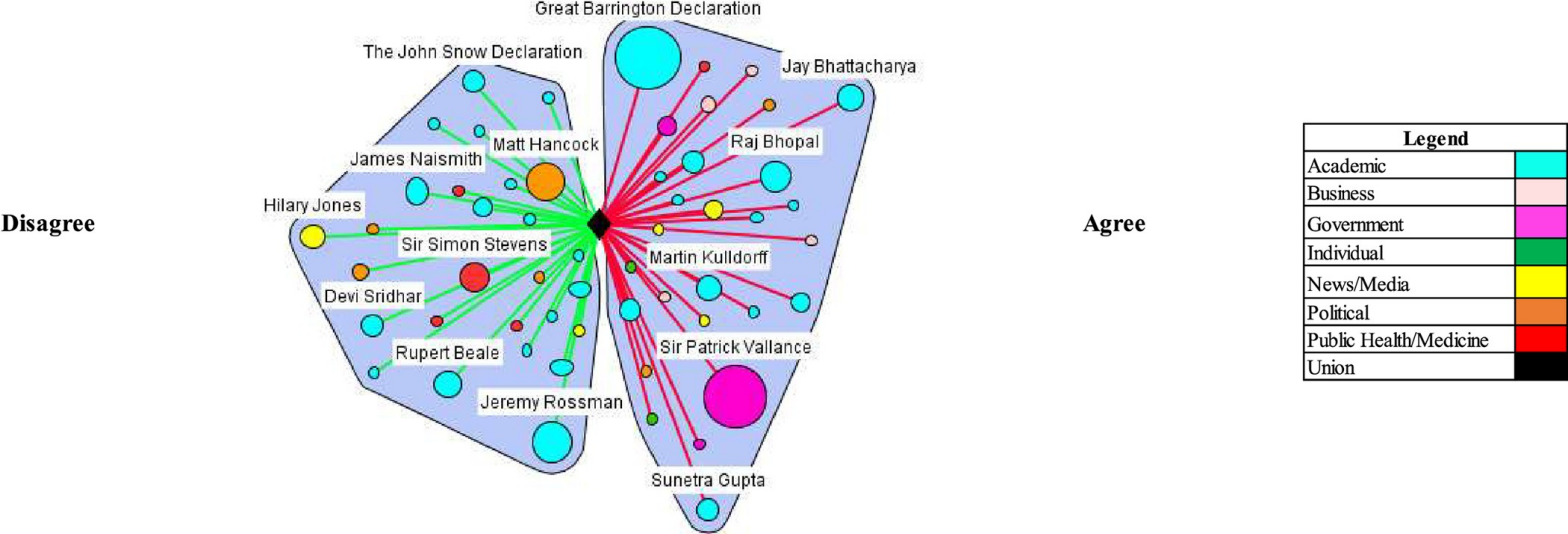
At risk populations can be protected in herd immunity approach (UK).

**Fig 6:**
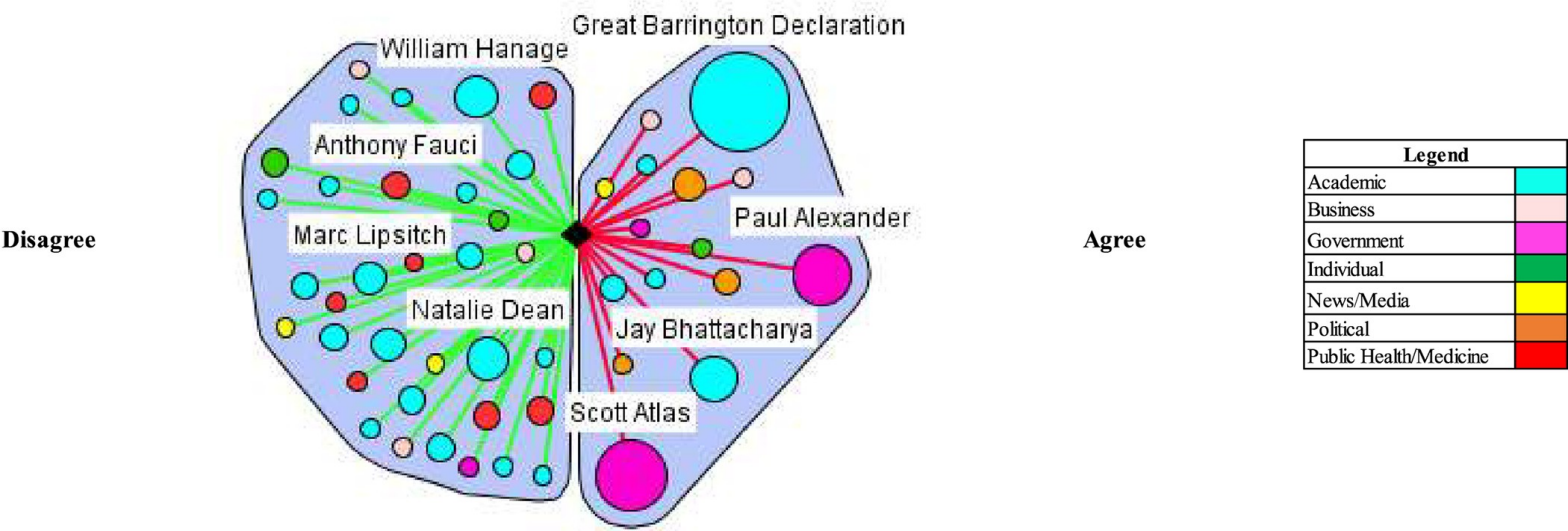
At risk populations can be protected in herd immunity approach (US).

Proponents argued that adopting a herd approach would not entail letting the disease spread to everybody in the population. They advocated for protecting at risk populations through isolation and other measures and letting groups low risk populations live normal lives to contract the virus and build immunity. For example, The Great Barrington Declaration and its authors vocally endorsed this position in the UK and US (Figs 5 and 6). The declaration argued for “focused protection” where “those who are at minimal risk of death to live their lives normally to build up immunity to the virus through natural infection, while better protecting those who are higher risk.” In the UK, Vallance made similar comments.

Most stakeholders argued it is not possible or ethical to segregate certain populations to protect them from the virus. For example, in the UK, Matt Hancock stated in the House of Commons in response to criticism from the authors of the Great Barrington Declaration that “[their] claim is that we can segregate the old and the vulnerable on our way to herd immunity. This is simply not possible.” In the US, academics questioned how proponents of natural herd immunity would protect at-risk groups. For example, Dr. William Hanage, an epidemiologist at Harvard University, stated: “[i]t [Great Barrington Declaration] offers the idea that we can just go back to normal, and it doesn’t say anything about how we’re going to do the most important part of it… You ought to give some indication of how you’re going to stop the grandparents being infected.” Academics, such as Dr. Marc Lipsitch at Harvard University, also critiqued the ability to identify high-risk groups.

### Concept #4: Healthcare services and capacity can be managed in herd immunity approach

**Fig 7:**
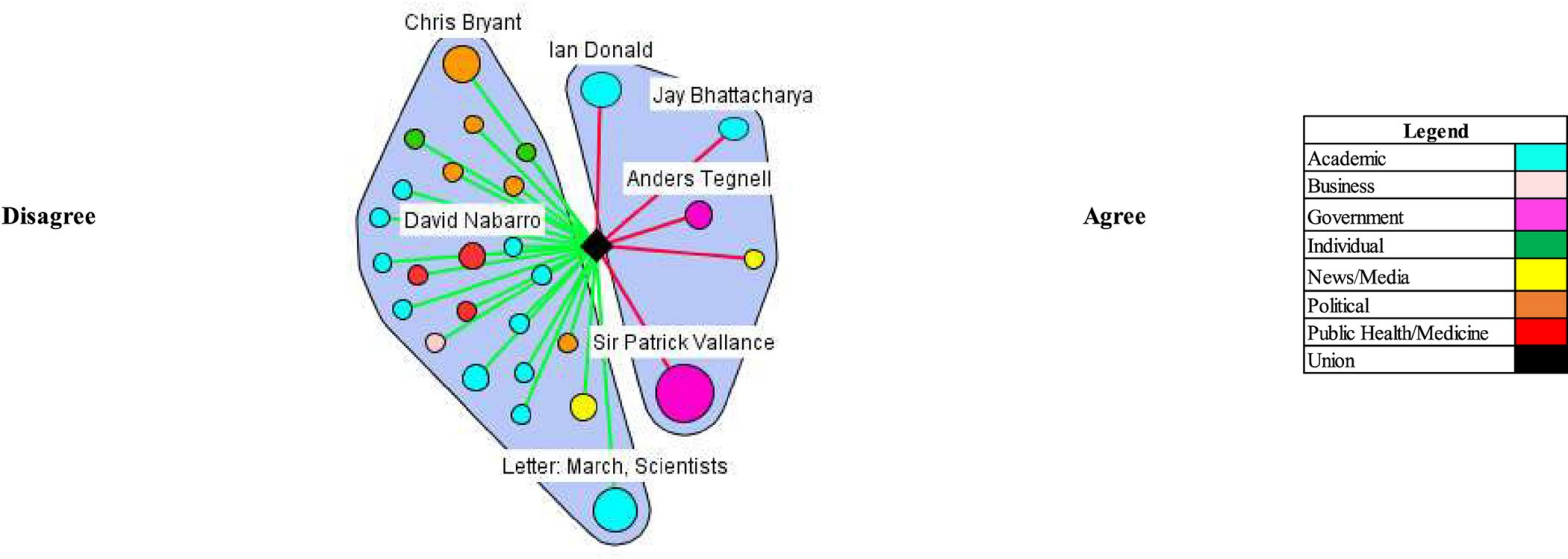
Healthcare services and capacity can be managed in herd immunity approach (UK).

**Fig 8:**
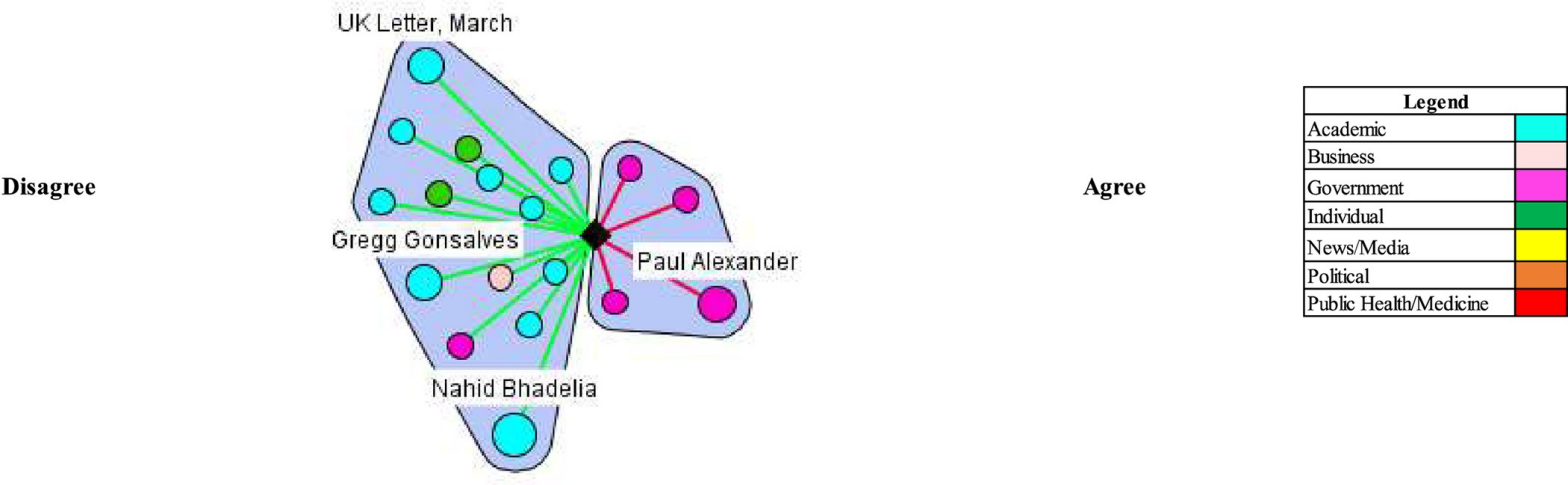
Healthcare services and capacity can be managed in herd immunity approach (US).

Several supporters of a herd immunity approach stated that it would be possible to minimize hospital admissions and ensure healthcare services are not overloaded in a herd immunity approach (Figs 7 and 8). For example, in the UK, Vallance stated in his remarks supporting herd immunity that “[w]hat we don’t want is everybody to end up getting it in a short period of time so we swamp and overwhelm [National Health Service] services.” In the US, Alexander argued in emails to Department of Health and Human Service colleagues that healthcare resources were ready for an influx of patients if needed: “hospitals are NOW geared, [personal protective equipment] in place, [intensive care units] bed are on the ready, doctors and nurses alert, the syndrome is crystalized…etc.”

Those disagreeing with the feasibility of allowing a herd immunity approach and managing healthcare resources argued that there is no plan for when health systems get overburdened. For example, scientists in the UK penned an open letter expressing this view as did Dr. Gregg Gonsalves at Yale, who spoke of hospitals being “overwhelmed” in the “hardest hit areas.”

## Discussion

Our findings show that, in sum, the media coverage around natural herd immunity portrayed a dismissal of the policy by the majority of academic and public health officials. However, considerable media attention was also given to a small, vocal, and heavily publicized coalition of scientists with prestigious credentials and prominent government advisors promoted and legitimized the strategy. As such, we observed evidence of false balance in the reporting among our sample of articles. Despite most actors disapproving from most stakeholder types, news coverage gave extensive attention to scientists promoting the strategy–notably, the Great Barrington Declaration and its authors. The false balance of reporting portrayed the natural herd immunity policy as one that was either accepted, or at least given reasonable consideration, by the scientific community. Public health officials in the media, however, were almost unanimously portrayed as against a herd immunity policy. As noted by Koehler, false balance in reporting can introduce controversy on uncontroversial subjects leading to distorted views among the public and actual behaviors such as reduced vaccine uptake [21]. Thus, false balance in reporting may have contributed to confusion, misinformed opinions, and reduced confidence and acceptance of mitigation measures. Indeed, social media discourse on health topics also illustrates how unscientific ideas can be aggressively promoted by likeminded participants thereby creating sharp divisions among the public and a heightened sense of perceived debate [22].

The central argument supporting natural herd immunity put forth by advocates was the false claim that it is possible to protect at risk populations and only allow low risk groups to contract COVID-19. All groups supporting a herd immunity approach noted that they did not advocate to allow the virus to spread unchecked [23]. However, the argument to isolate certain populations was countered by others as a false option. Indeed, it is not feasible to shield such groups when allowing other groups to become infected [6]. First, it is not possible to fully identify at risk groups [12]. Many people in both the UK and US have undiagnosed health conditions that presents a higher risk of suffering or death from COVID-19 [24]. Reliance on younger people experiencing lower mortality does not incorporate the suffering or long-term respiratory damage from infection [7]. Second, populations perceived at low-risk work in frontline or healthcare jobs and interact often with those who are deemed high risk and can pass on the infection to such groups. For example, outbreaks in long term care homes during the pandemic were often introduced unintentionally despite strict precautions and led to excess deaths [25, 26].

Our findings illustrate how the concept of herd immunity was given prominence as a means of countering the spread of COVID-19 in the UK and US media. Notably, as mentioned, when public health officials were featured in the media, in either country, their voices were nearly unanimous in disagreement with the herd immunity concept. In contrast, the concept was debated among government officials, academics and political figures. As such, these disagreements likely contributed to an increased politicization of the topic where the evidence surrounding the realistic impact of herd immunity implementation might have struggled to gain traction among audiences with contrastingly political allegiances. Further, these disagreements might have led to divisions among the public and their uptake of mitigation measures [27] such as masks, social distancing, and lockdowns. In other circumstances, comments to the media from academics regarding natural herd immunity may have been reported out of context or the comments did not properly articulate the intended meaning. For instance, Dr. Graham Medley wrote to The Lancet stating that his comments to media were misrepresented in a published research article [28].

A limitation of our methods is that we applied filters to reduce the number of articles in our search strategy for project feasibility. Thus, our sample of articles is not exhaustive and there are likely additional comments from other stakeholders that were not included in our study. However, the number of articles collected led to saturation in comments retrieved. A great quantity of articles would not have identified other concepts to include in our study. The study also did not intend to collect every statement, but rather the dominant discourse narratives in high profile outlets from recognizable stakeholders, which our methods accomplished.

Our findings underscore the need for greater appreciation of potential harm of media representations that contain false balance. This is particularly important in the context of unprecedented misinformation during a public health emergency [29]. As current discussion of the safety and efficacy of vaccines for COVID-19 and timelines for lifting social distancing measures reveal, problematic news media coverage during the COVID-19 pandemic is likely not limited to discussions of natural herd immunity. Nor, unfortunately, will this pandemic be a one-off event, underscoring the need now to address these concerns through future research. Scientific debate deserves coverage in the media–but if media outlets do not take precautions, it may lead to unnecessary controversy or misinformed portrayal in public discourse. Continual efforts must be made to mitigate false balance in emergency public health reporting whether that be from journalists themselves or from the public interpreting the news.

## Supporting information

S1 Dataset United Kingdom coding events list. (CSV)

S2 Dataset United States coding events list. (CSV)
